# A comparison of the differences in the way parents and grandparents interact with children and their effects on children’s creative performance

**DOI:** 10.3389/fpsyg.2022.1066524

**Published:** 2022-12-21

**Authors:** Ye Lu, Yilai Pei, Weiguo Pang

**Affiliations:** ^1^Youth Research Data Center, Shanghai Youth College of Management, Shanghai, China; ^2^Institute of Developmental and Educational Psychology, School of Psychology and Cognitive Science, East China Normal University, Shanghai, China

**Keywords:** parent–child interaction, grandparent–grandchild interaction, divergent thinking, convergent thinking, intergenerational parenting

## Abstract

As grandparents’ involvement in parenting becomes more common, it is valuable to understand the differences between grandparenting and parenting and how these differences affect children. To elucidate the differences between grandparenting and parenting and their effects on children’s creativity performance, children’s performance on creativity tasks after grandparent–child interactions and parent–child interactions were compared, and the behavioral differences between grandparents and parents when interacting with children were discussed. In this study, grandparents and parents were asked to interact with children separately, and creativity performance was measured before and after adult-child interactions. The results showed that children’s creative performance improved significantly after parent–child interactions, while there was little change after grandparent–child interactions. In addition, according to parental investment theory, parents provided children with more cognitive and interpersonal resources during the interaction compared to grandparents.

## Introduction

### Grandparental involvement changes the family environment in which children grow up

Family environment is usually considered to be an important factor influencing children’s creativity (Lew and Cho, 2013; Zhang et al., 2018; Jankowska et al., 2020), and previous studies have shown that some characteristics of families, such as socioeconomic status (Miller and Gerard, 1979; Dai et al., 2012; Runco, 2014), parenting styles (Fearon et al., 2013; Mehrinejad et al., 2015), parent–child relationship (Zhang et al., 2018), and parental involvement (Wright and Wright, 1986; Liu et al., 2013; Robinson et al., 2013; Kim and Hill, 2015) are important predictors of children’s creativity. For example, research has found a positive correlation between parental creativity and children’s creativity (Fearon et al., 2013), and a moderate association between their beliefs and mindsets about creativity (Karwowski et al., 2022), suggesting that family environment or interaction with parents may contribute to children’s creative mindset and creativity (Torrance and Hansen, 1965; Hass et al., 2017; Karwowski et al., 2022).

Nowadays, grandparenting is increasingly being adopted as a preferred solution to reduce the need for formal child care and to free mothers from child care on behalf of working parents (Shakya et al., 2012; Hayslip et al., 2019), which has led to some changes in the family environment in which children grow up. In the United States, 3 million grandparents are reported to claim primary childcare responsibility for grandchildren under the age of 18 (Generations United, 2015). In the United Kingdom, the Grandparents Association estimates that more than 13.5 million grandparents provided approximately 60% of childcare in the United Kingdom in 2006 (Tan et al., 2010). In South Korea, more than 60% of working mothers use kinship parenting, with grandparents being the most supportive (Lee and Bauer, 2010). In China, nearly 80% of Chinese families have grandparents involved in raising their grandchildren, according to a 2017 study by the Family Education Committee of the Chinese Education Association (Yue, 2018). With the popularity of grandparenting, there has been a growing body of research on intergenerational parenting, in which a growing body of research has shown that in addition to parents, grandparents play an important role in the lives of children and adolescents in the family (Attar-Schwartz et al., 2009; Li et al., 2019; Nedelcu and Wyss, 2020).

### The advantages and disadvantages of grandparental involvement for children development

On the one hand, help from grandparents, both past and present, is highly associated with maternal fertility success. A review by Sear and Mace (2008) based on 45 articles of literature revealed that the presence of a grandmother or maternal grandmother increased the chances of successful survival of grandchildren by 53%–69%. Adolescents perceive that emotional intimacy with parents and grandparents may reduce their adaptation difficulties, whereas adolescents’ emotional intimacy with parents moderates their emotional intimacy with grandparents and adolescents’ adjustment difficulties (Attar-Schwartz, 2015). For those families that are divorced or remarried, the role of grandparents becomes more pronounced. It was found that there was no difference in the level of grandparent involvement across family structures, but the positive role of grandparents was more pronounced among adolescents in single-parent and step-parent families (Dunifon and Kowaleski-Jones, 2007; Attar-Schwartz et al., 2009). That is, the higher the involvement of grandparents, the less emotional problems, and the more pro-social behavior of the children, which seems to suggest that grandparents, as an alternative resource to parents, can play a compensatory role to some extent (Attar-Schwartz and Khoury-Kassabri, 2016).

However, on the other hand, according to other studies, grandparenting is detrimental to children’s cognitive development. Marks (2007) suggested that living with grandparents is associated with lower achievement outcomes of children, and grandparental psychological control was positively linked to children’s behavioral problems for highly reactive children (Gao et al., 2022). Pilkauskas and Martinson (2014) found that living with grandparents was more common in less advantaged families. Hancock et al. (2016) also supported that the negative impact of low grandparent education on grandchildren was more pronounced in families with more frequent contact, as these families are more likely to need grandparent support. It appears that research is inconclusive as to whether grandparental involvement in parenting is beneficial to their grandchildren’s development.

### Will grandparental involvement affect children’s creativity development?

Although there are plenty of studies on the effects of intergenerational parenting on children’s cognitive, emotional, and ability development, few studies have focused on the effects of intergenerational parenting on children’s creativity. Creativity is often defined as the development of original ideas that are useful or influential (Runco, 2004). Researchers held that creative thinking is a divergent process based on a great deal of experience and learning (Amabile, 1988; Kwaśniewska et al., 2018). Parents may directly provide their children with some knowledge and experience in their daily relationship or interaction with their children, or they may set some positive examples to promote children’s creativity (Jankowska and Gralewski, 2022).

In order to foster children’s creativity, both nuclear and intergenerational families should try to create a harmonious family atmosphere and provide more opportunities for children to engage in creative activities (Gaynor and Runco, 1992). Meanwhile, the way the main caregivers in the family get along with their children is extremely critical. Wright (1987) reported that both parents and grandparents can influence children’s creativity by modeling creativity in their daily behavior. Pang et al. (2020) found that a three-generation family structure, especially grandparent-dominated families, is not conducive to children’s creativity development.

Realistically, the current trend of intergenerational parenting is difficult to change, yet given the possible drawbacks of grandparenting, such as overindulgence (Adler, 1964) that may lead to restricted development of excessive self-centeredness (Jiao et al., 1986) and autonomy (Deci and Ryan, 2012) in grandchildren, which reduces children’s creativity, examining grandparenting and paternal parenting differences and the impact of such differences on children’s creativity development would be valuable. Therefore, the present study would like to explore the differences between parents and grandparents in their interaction with children, and how such differences affect children’s creativity.

### Differences in resources that parents and grandparents provided to children from the perspective of parental investment theory

From the perspective of parental investment theory (Hertwig et al., 2002), the atmosphere created by parents and all the attitudes and behaviors displayed by parents in the parenting process can be considered as “family resources” which may influence children’s creative performance.

According to Hertwig et al., there are at least three types of family resources: (1) material resources, such as money for food, health care, and higher education; (2) cognitive resources, such as intellectual stimulation, training, and guidance; and (3) interpersonal resources, such as attention, time, love, affection, and general encouragement. The three types of resources can be distinguished as follows. Material resources aim to provide a good living and educational environment for children, cognitive resources are reflected in children’s intellectual development through parents’ intellectual and time investment, and interpersonal resources are reflected in parents’ support for children at the level of non-intellectual factors (mainly emotional). In view of this, parents’ economic base, cognitive level, personality traits, and emotional values can be considered as resources. Conger and Donnellan (2007) reported that if parents, especially mothers, have a high level of education and create a good environment inside and outside the home, they can provide children with rich social resources that can promote children’s cognitive abilities and social skills. In addition, various strengths of resources associated with parents play a positive role in the development of children’s creativity. Existing research suggests that parents of highly creative individuals rarely control their children’s behavior through dogmatic rules. Instead, they often provide their children with values and encourage them to make their own decisions based on these values (Dacey, 1989). Sternberg and Lubart (1995) argues that parents of highly creative individuals generally have diverse interests and open minds, and invest more time and money in their children. It has been suggested that parents of highly creative individuals in the family are generally able to support their children’s interests, tolerate their failures, and set good role models for them (Gute et al., 2008). According to a study based on Korean elementary school students, there was a significant positive correlation between parental acceptance and children’s creative personality (Lim and Smith, 2008). Other studies have shown that creativity is positively correlated with parental encouragement. Parents of highly creative individuals often compliment their children, spark their interest, encourage innovation, and engage in their learning process (Robinson et al., 2013; Sen and Sharma, 2013). All the above can be seen as rich parental resources that parents invest in highly creative individuals.

In the parental investment framework, grandparents’ participation is also recognized as a kind of family resource. Coall and Hertwig (2010) conceptualized grandparental investment through Trivers’ (1972) concept of parental investment. It refers to the resources that grandparents transfer to their grandchildren, or resources that benefit their grandchildren, and the exact opportunity cost. Grandparental resources are multidimensional in nature, including practical help, food production, finances, time in the form of childcare, or the emotional support provided by a listening ear, all of which are grandparent investments. Grandparents can play a variety of active roles, such as storytellers, family historians, mediators or counselors, and people who encourage children, teach them knowledge and skills, and even some life wisdom (Michalski and Shackelford, 2005; Gurven and Schniter, 2010). In a real-life setting, however, the results of grandparent involvement in grandchildren’s parenting can be double-edged. A typical example is that one of the most common behaviors of grandparents engage in with their grandchildren is watching television (Höpflinger and Hummel, 2006). At its best, watching television together can educate children and facilitate intergenerational dialog. Today, however, watching television leads to a sedentary lifestyle, which results in frequent physical conditions in children raised by grandparents: the body mass index (BMI) of grandchildren is positively correlated with the BMI of their grandparents. Also, the higher the grandparents’ BMI, the lower the grandparents’ activity level, and the lower the grandparent’s activity level, the more time the grandchildren spent watching television (Polley et al., 2005). King and Elder (1998) found that grandparents with low levels of education were more actively and deeply involved in their grandchildren’s upbringing. Relative to highly educated grandparents, low-educated grandparents were more likely to agree that it was important for their opinions to be able to influence others in the family. Fuller-Thomson and Minkler (2001) noted that grandparents who acted as “surrogate parents” and raised their grandchildren almost full-time were less educated than those who never or occasionally participated in parenting. Apparently, grandparents involved in parenting activities seem to be more inclined to invest in material resources (ensuring children’s food and clothing) and some interpersonal resources. In addition, they tend to overinvest in material resources and underinvest in cognitive resources. Thus, the first hypothesis is, *when parents and grandparents interact with children separately, parents can provide significantly more cognitive and interpersonal resources than grandparents.*

### How differences in family resources provided by the parents and grandparents affect children’s creativity

Intergenerational comparative studies on child rearing have shown that there are several differences in child rearing between grandparents and parents, and such differences can have different effects on children. A study that examined differences in attitudes and perceptions of child-rearing among grandparents and parents showed that grandparents placed more emphasis on behavioral training and traditional filial education for their children, while parents placed more emphasis on their children’s independence, autonomy, and creativity (Liu, 1995). China Family Development Report (NHFPC of the PRC, 2016) states that grandparenting focuses on children’s daily life, such as eating, dressing, and physical health, while parents paid more attention to children’s discipline and education, such as regulating children’s behaviors and helping them develop good habits. In addition, a study comparing the differences in parenting perceptions between grandparents and parents in a three-generation family concluded that grandparents place more importance on their children’s physical health, while parents place more importance on their children’s learning and creativity (Lin, 1999). Li et al. (2019) examined the relationship between grandparenting style and grandchildren’s emotions and behaviors while controlling for parenting style, and showed that grandparenting style, especially grandparental care and overprotection, was positively related to emotional and behavioral problems, while parental care was negatively related to children’s externalizing behavioral problems.

It is therefore clear that parents and grandparents have their own priorities in terms of parenting concepts and behaviors. Relatively, parents are more flexible and treat them with more equality and respect, which is certainly more conducive to the development of children’s creativity, while grandparents’ parenting behaviors are more dogmatic and stricter, but more doting, which does not seem to be as conducive to the development of creativity.

In terms of the family resources involved in intergenerational parenting, a family’s material resources are not likely to change much as most Chinese grandparents consider themselves to be child-rearing assistants and the primary responsibility for child rearing still lies with the parents (Leung and Fung, 2014). Considering that grandparental involvement is more about time and effort rather than financial support, grandparental involvement in child rearing will not affect the total income of the extended family generally, i.e., the household income is relatively constant. However, it is undeniable that the current cohort of Chinese grandparents, most born before 1970, represents a relatively more traditional and perhaps less educated group of Chinese (Xu and Chi, 2015; Qiu and Shum, 2022). Accordingly, when grandparents are involved in parenting activities as alternative or complementary resources for parents, their ability to provide children with cognitive resources (e.g., grandparents’ educational level and intellectual stimulation) and interpersonal resources (e.g., grandparents’ positive attention and emotional encouragement), which are crucial for children’s creative development, may be significantly reduced. Differences in parenting behaviors between parents and grandparents may lead to qualitative differences in children’s development of creativity. Specifically, interaction with parents, but not grandparents, is associated with increased creativity. Thus, the second hypothesis is, children’s creativity performance will increase after interacting with their parents, but not after interacting with their grandparents.

## Materials and methods

### Participants

Participants were 74 pairs of elementary school students and their parents and grandparents, including 35 girls (47.3%), from three public elementary schools in Fengxian District, Shanghai. The mean age of the participants was 9.51 years, with a standard deviation of 1.19 years. Among all families participating in the experiment, 72.9% of the parents had college or higher education, but only 6.8% of the grandparents had college or higher education, mostly in elementary (43.2%) and junior high school (20.3%). 90.5% of parents had stable jobs, with white-collar occupations predominating, while 82.4% of grandparents had retired, and the vast majority were blue-collar workers before retirement. Each group of families (one student, one parent, and one grandparent) signed an informed consent form prior to the start of the study, in which the students were signed by their parents, and their verbal consent was obtained. Upon completion of all study tasks, each group of families will be paid 100 RMB.

### Experimental design

A field experiment was used to control for parent–child interactions and grandparent–child interactions in a three-generation family, so as to capture the behavior of parent-child and grandparent-child interactions in a real environment and thus improve the ecological validity of this study.

A 2 × 2 mixed experimental design was used. The within-group variable was “whether there was interaction” (specifically, “before interaction” and “after interaction”), and the between-group variable was “interaction with which family member” (specifically, “parent–child interaction” and “grandparent-grandchild interaction”). The dependent variable was children’s creativity.

### Measures

#### Children’s creativity

Previous research has shown that creativity is best measured using multiple instruments (Long and Plucker, 2015), so this study selected the three most commonly used instruments for measuring creativity, namely the Torrance Tests of Creative Thinking-Figural (TTCT-Figural; Torrance, 1974), the Alternative Uses Test (AUT; Kudrowitz and Dippo, 2013) and insight problem solving (Chuderski and Jastrzębski, 2018). Considering the young age of the participants, the first tool chosen was the Alternative Uses Test (AUT), which is a relatively simple but reliable and efficient assessment of verbal divergent thinking—a key indicator of creative potential (Runco and Acar, 2012). Students were asked to write down as many novel uses of two common items (e.g., pencils, socks) as they could in 3 min, the more the better. The second tool was the Torrance Test of Creative Thinking-Figural Test subscale (TTCT-Figural; Torrance, 1974). The TTCT is a widely used and psychologically reliable test of creativity (Kim, 2011). The original TTCT has two versions: the TTCT-Figural and TTCT-Verbal, each with two parallel forms, Form A and Form B (Torrance, 2008). Since the AUT scores were used to represent verbal creativity, only Form B was used to measure nonverbal creativity. In this task, participants were asked to complete simple and abstract lines into meaningful pictures and then to name the pictures.

Creative problem solving requires generating a variety of solutions or hypotheses through divergent thinking, but also requires individuals to select appropriate problem-solving strategies based on task requirements, rely on cognitive monitoring to break through thinking stereotypes, and ultimately generate optimal creative task solutions. Therefore, insight tasks were added as an indicator of convergent thinking in order to examine the influence of parental and grandparental factors more comprehensively on children’s creativity. The insight tasks were chosen to be as relatively simple as possible to match the cognitive ability level of elementary school students, such as “If you have black socks and brown socks in your drawer, mixed in the ratio of 4:5, how many socks will you have to take out to be sure of having a pair of the same color?” (Fleck, 2008; Jia et al., 2009; Fleck and Weisberg, 2013; Trina et al., 2013; Wang, 2017).

Creativity was evaluated on the following specific indicators: originality and elaboration of TTCT-Figural, originality, and fluency of AUT, and insight problem-solving scores. Originality of TTCT-Figural, elaboration of TTCT-Figural, originality of AUT, and fluency of AUT were assessed by using Consensual Assessment Technique (CAT, Amabile, 1983; Kaufman et al., 2009), obtained by three trained raters, all of whom were PhD candidates in the field of creativity research, with rating consistency coefficients of 0.71 and 0.97. The CAT is a powerful tool used by creativity researchers to rate the creativity of a set of creative products (e.g., stories, paintings, poems, etc.) by several expert judges who must be experts in the field (Kaufman et al., 2010). Since CAT is not based on any particular theory of creativity, its validity (which has been well-established empirically) does not depend on the validity of any particular theory of creativity. For this reason, it has been called the “gold standard” for creativity assessment (Baer and McKool, 2009). Insight problem solving consisted of four questions, with the total score being the sum of all correct responses, with a minimum score of 0 and a maximum score of 4.

#### Family resource

In this study, video observation and post-coding were used to measure how children interacted with their parents and grandparents. Adult-child conversations and interactions were coded through observation methods of event sampling and synthetical evaluation within the framework of parental investment theory (Hertwig et al., 2002; Hou et al., 2005). Coding of what parents say includes inquiry (e.g., questioning), prompting instruction (e.g., guiding), exemplary instruction (e.g., exampling and explanation), positive feedback (e.g., affirmation, encouragement, and approval), negative feedback (e.g., criticism, direct negation, and euphemistic negation). Coding of parental behavior included demonstrative instruction (e.g., gesturing with hands, writing out answers instead of the child), positive feedback (e.g., nodding, thumbs up), etc. The synthetical evaluation method was used to evaluate the relationship between children and adults (i.e., parents or grandparents) in terms of dimensions such as body distance, intimacy, and emotional state. According to the parental investment theory, adult guidance is a cognitive resource, feedback is an interpersonal resource, and inquiry is not strictly considered a cognitive resource (see Table 1 for specific coding).

**Table 1:** Video coding dimensions and specific indicators for parent–child interaction and grandparent–child interaction.

Coding dimension	Specific indicators	Scoring	Resource type
Duration of interaction	1. Effective interaction time	Objective scoring, timing	/
Event code (adult)	2. Inquiry (e.g., what are you drawing?)	Objective rating: counting times, 1 point per time	/
	3. Prompting instruction (e.g., *think about it, what did we do when you are bitten by mosquitoes last time?*)	Objective rating: counting times, 1 point per time	Cognitive resources (instructing)
	4. Exemplary instruction (e.g., *newspapers can also be used as hats…*)	Objective rating: counting times, 1 point per time	Cognitive resources (instructing)
	5. Ability of problem solving	Subjective rating: 1–5 scale	Cognitive resources (cognitive abilities)
	6. Positive feedback (e.g., *this is a great idea!*)	Objective rating: counting times, 1 point per time	Interpersonal resources (encouragement)
	7. Negative feedback (e.g., *what are you drawing? I cannot understand it*)	Objective rating: counting times, 1 point per time	Interpersonal resources—Negative Scoring (discouraging)
State coding (adults and children)	8. Body distance	Subjective rating: 1–5 scale	Interpersonal resources (attention)
	9. Parent–child/grandparent–child intimacy	Subjective rating: 1–5 scale	Interpersonal resources (love)
	10. Adult’s emotional state	Subjective rating: 1–5 scale	Interpersonal resources (affection)
	11. Child’s emotional state	Subjective rating: 1–5 scale	/

The coding was performed independently by two trained coders, both of whom are PhD candidates in the field of creativity research. The process was as follows: (1) converting all video materials (296 in total) into textual materials in the form of dialogs; (2) using parents’ investment in their children as a theoretical framework, identifying the behavioral codes to be recorded in parent–child interactions and grandparent-child interactions, and determining the operational definitions of each behavioral code; and (3) event coding, which was mainly based on what the adults (i.e., parents and grandparents) said, with the coding method being objective scoring. For example, the adult instructs or prompts the child several times during the interaction. “Think about what socks can be used for at Christmas.” “What are the little dolls made of?.” The adult scored 1 point for each reminder; (4) state coding, based primarily on nonverbal performance (e.g., physical distance, emotional attitude, etc.), was scored on a 5-point scale. The coding alpha coefficient ranged from 0.71 to 0.97, which is relatively good.

### Procedure

A group of participants, “a child and an adult,” were experimented one-on-one by an experimenter. Each child participated in the experiment twice, one with his parents and one with his grandparents. The order of the parent and grandparent participation in the study was randomly selected based on the timing of the participants, meaning that some children participated in the experiment with their grandparents first and some children participated with their parents first, with approximately 2 weeks between two experiments.

**Figure 1:**
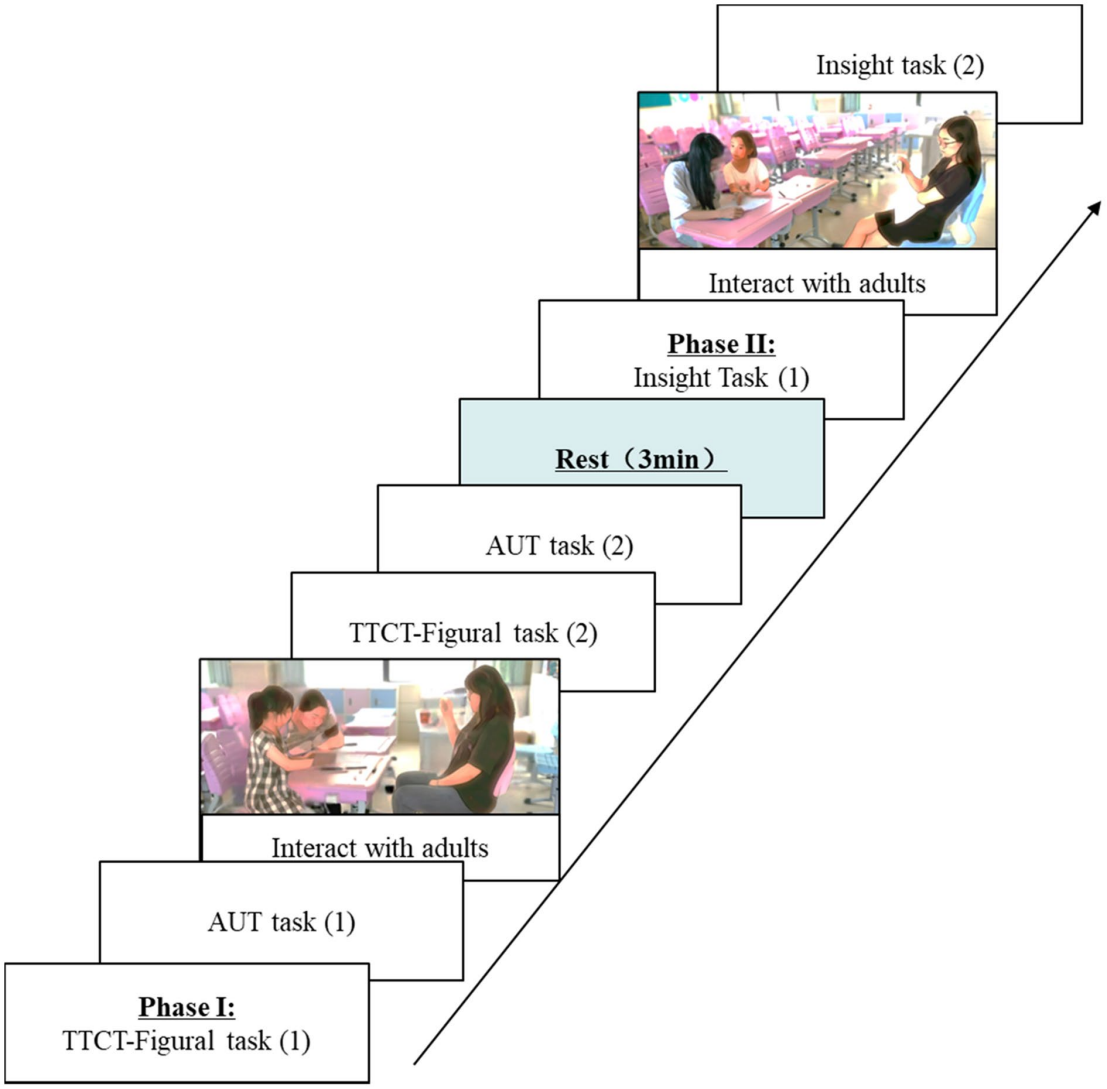
A single complete experimental procedure (one child, single experiment).

In each experiment, children were asked to complete two sets of creativity tests of equal difficulty (Form AB and Form CD). The procedure was identical for both experiments (see Figure 1 for details). Specifically, the first experiment used FormAB, with different questions on FormA and FormB, but the same type of test questions and task difficulty, and the order of tests on FormA and FormB was randomized; the second experiment used FormCD, with different questions from the first experiment, but the same type of questions and task difficulty, and the order of tests on FormC and FormD was also randomized. The order in which parents and grandparents participated in the study was randomly selected based on the timing of the participants, meaning that some children participated in the experiment with their grandparents first and some children participated in the experiment with their parents first, with approximately 2 weeks between the two experiments.

In each complete experiment, once with a parent and once with a grandparent, children were asked to complete (1) a set of TTCT-Figural tasks and AUT tasks in Form AB or Form CD, each with a time limit of 3 min; (2) grandparent–child interaction or parent–child interaction, each with a time limit of 3 min, for a total of 3 tasks; (3) another set of TTCT-graphic tasks in Form AB or Form CD of the same difficulty TTCT-Figural task and AUT task with a time limit of 3 min each for a total of 3 tasks; (4) a set of insight problem solving in form AB or form CD with a time limit of 3 min each for a total of 4 tasks; (5) grandparent–child interaction or parent–child interaction with a time limit of 5 min; and (6) another set of insight problem solving in form AB or form CD with the same difficulty with a time limit of 3 min each for a total of 4 tasks.

Both processes of grandparent-grandchild interaction or parent–child interaction were recorded in video form for later behavioral coding by the researchers. The instructions for grandparent–grandchild interaction or parent–child interaction are as follows. “Dad/Mom/Grandpa/Grandma, this is a task that your child has just completed. Please have a look or listen to what he says. You can have a conversation or interaction about this task, such as giving him/her some feedback or comments. During this process, we will take a video of up to 5 min. Thank you!” Each group of participants will be randomly divided into four experimental conditions, and the actual experimental sequence is shown in Table 2.

**Table 2:** Distribution of experimental participants.

	Form AB		Form CD		Total
	A-B	B-A	C-D	D-C	
Parent–child interaction	22	18	14	20	74
Grandparent–child interaction	17	17	23	17	74
Total	39	35	37	37	

### Data analysis

SPSS 22.0 is used to analyze the data.

## Results

To test hypothesis 1, a paired-samples *t*-test was conducted on the resources given by parents and grandparents during the interaction (i.e., cognitive and interpersonal resources), and the results showed that there were significant differences between parent–child interactions and grandparent–grandchild interactions in a family (see Table 3). Specifically, parents would ask more questions and provide more guidance to their children through prompting and exemplification during their interactions with their children. At the same time, parents themselves would have better problem-solving skills than grandparents, which belong to the cognitive resources that adults can provide to children. As for interpersonal resources, children were closer and more intimate when interacting with their parents. In addition, children also had significantly better emotional states during interactions with parents than with grandparents, and parents had significantly better emotional states during interactions than grandparents. There was a significant positive correlation between adult and child affect during the interaction (*r*_parents × children_ = 0.767^**^, *r*_grandparents × children_ = 0.495^**^).

**Table 3:** Comparison of cognitive and interpersonal resources given by parent–child interaction and grandparent–child interaction (paired-sample *t*-test).

	Scoring index of interaction	*M*_*Parent*–*child interaction*_	*SD*_*Parent*–*child interaction*_	*M*_*Grandparent*–*grandchild interaction*_	*SD*_*Grandparent*–*grandchild interaction*_	*t*	*p*
Duration of interaction	Effective interaction time	261.86	69.46	223.83	82.11	5.119^**^	0.000
Not coded	Inquiry	3.87	3.87	2.70	2.77	4.318^**^	0.000
*Cognitive resources*	Prompting instruction	*1.51*	*2.08*	*0.30*	*1.26*	7.695^**^	0.000
	Exemplary instruction	*1.85*	*2.03*	*0.65*	*1.18*	6.686^**^	0.000
	Ability of problem solving	*2.06*	*1.08*	*1.15*	*0.62*	9.991^**^	0.000
*Interpersonal resources*	Positive feedback	*0.41*	*0.90*	*0.36*	*0.79*	0.640	0.523
	Negative feedback	*0.21*	*0.57*	*0.32*	*0.79*	−1.474	0.143
	Body distance	*3.38*	*0.77*	*2.93*	*0.90*	5.760^**^	0.000
	Intimacy	*3.40*	*0.65*	*2.71*	*0.76*	11.013^**^	0.000
	Adult’s emotional state	*3.32*	*0.59*	*2.91*	*0.55*	7.26^**^	0.000
Not coded	Child’s emotional State	3.17	0.71	2.83	0.69	5.95^**^	0.000
	Education level	4.05	0.96	1.95	1.06	12.669^**^	0.000

* *p* < 0.05;

** *p* < 0.01.

**Figure 2:**
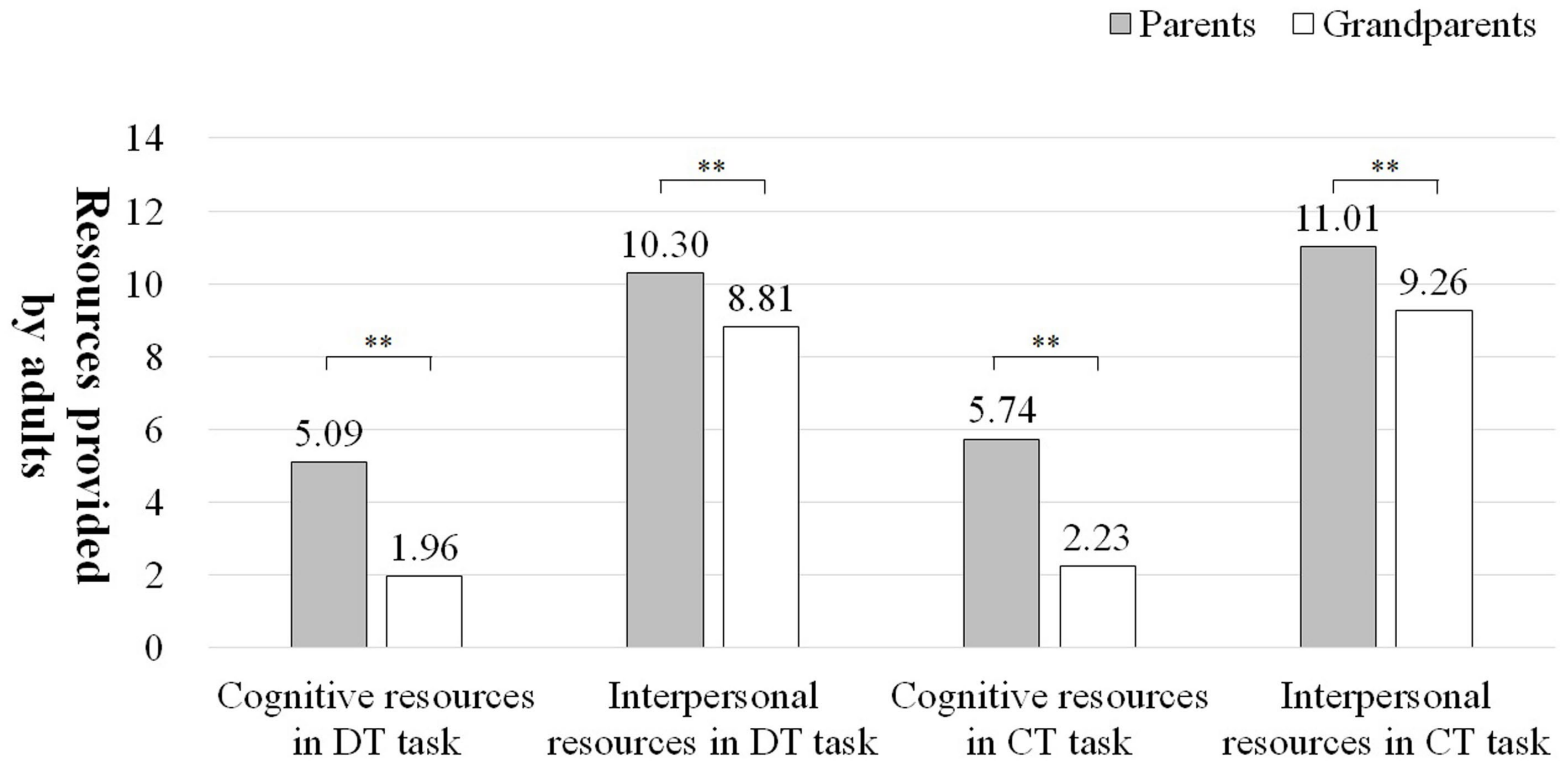
Comparisons of the cognitive resources and interpersonal resources given to children by parents and grandparents. **indicates that the difference is significant at the 0.01 level.

According to parental investment theory, prompting instruction and exemplary instruction provided by adults in the interaction, as well as adults’ problem-solving skills, are cognitive resources. The sum of positive feedback, body distance, intimacy, and adult’s emotional state minus negative feedback are interpersonal resources. During the interaction, parents gave more resources to their children compared to grandparents, both in terms of cognitive and interpersonal resources (*t*_cognitive resources in DT task_ = 6.180^**^, *t*_interpersonal resources in DT task_ = 5.024^**^, *t*_cognitive resources in CT task_ = 5.703^**^, *t*_interpersonal resources in CT task_ = 4.517^**^, see Figure 2). Thus, as described in Hypothesis 1, parents can provide more cognitive and interpersonal resources than grandparents in their interactions with their children.

The results of the correlation analyses (Table 4) indicated that the level of cognitive and interpersonal resources provided by parents or grandparents was correlated with their level of education, that is, the higher the level of education of the adults with whom the child interacted, the more family resources they could provide. Therefore, it was necessary to include adults’ education level as a control variable in the statistical analyses presented next.

**Table 4:** Correlation analysis between adults’ education level and resources provided by them.

	1	2	3	4	5	*M*	*SD*
1. Adults’ education level	1					3.00	1.46
2. DT tasks-cognitive resources	0.433^**^	1				3.53	3.45
3. DT tasks-interpersonal resources	0.390^**^	0.295^**^	1			9.56	1.95
4.CT tasks-cognitive resources	0.502^**^	0.754^**^	0.210^*^	1		3.98	4.12
5. CT tasks-interpersonal resources	0.391^**^	0.286^**^	0.725^**^	0.289^**^	1	10.13	2.51

The education level is coded as follows: 1 = Primary school education; 2 = Junior high school education; 3 = high school education; 4 = College education; 5 = Bachelor’s education; 6 = Master’s degree education; 7 = PhD education. **indicates that the correlation is significant at the 0.01 level. *indicates that the correlation is significant at the 0.05 level.

In the experiment, adult-child interactions were limited to 5 min (i.e., 300 s), but in terms of effective duration of parent–child and grandparent–grandchild interactions, effective parent–child interactions were significantly longer than grandparent–grandchild interactions between the two divergent thinking tasks (i.e., DT tasks, including TTCT-Figural and AUT), and between the two convergent thinking tasks (i.e., CT tasks, used to address insight problems).

**Figure 3:**
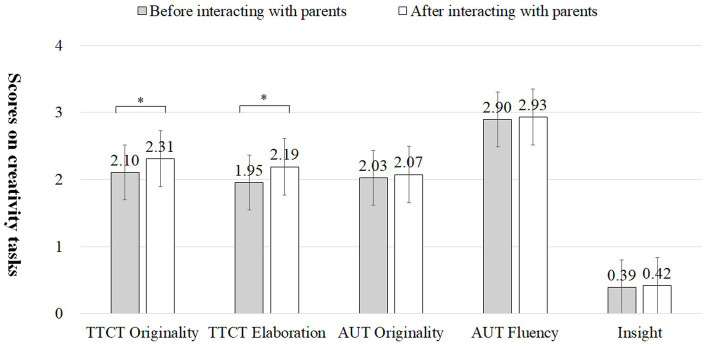
Differences in children’s scores on creativity tasks before and after interaction with parents.

**Figure 4:**
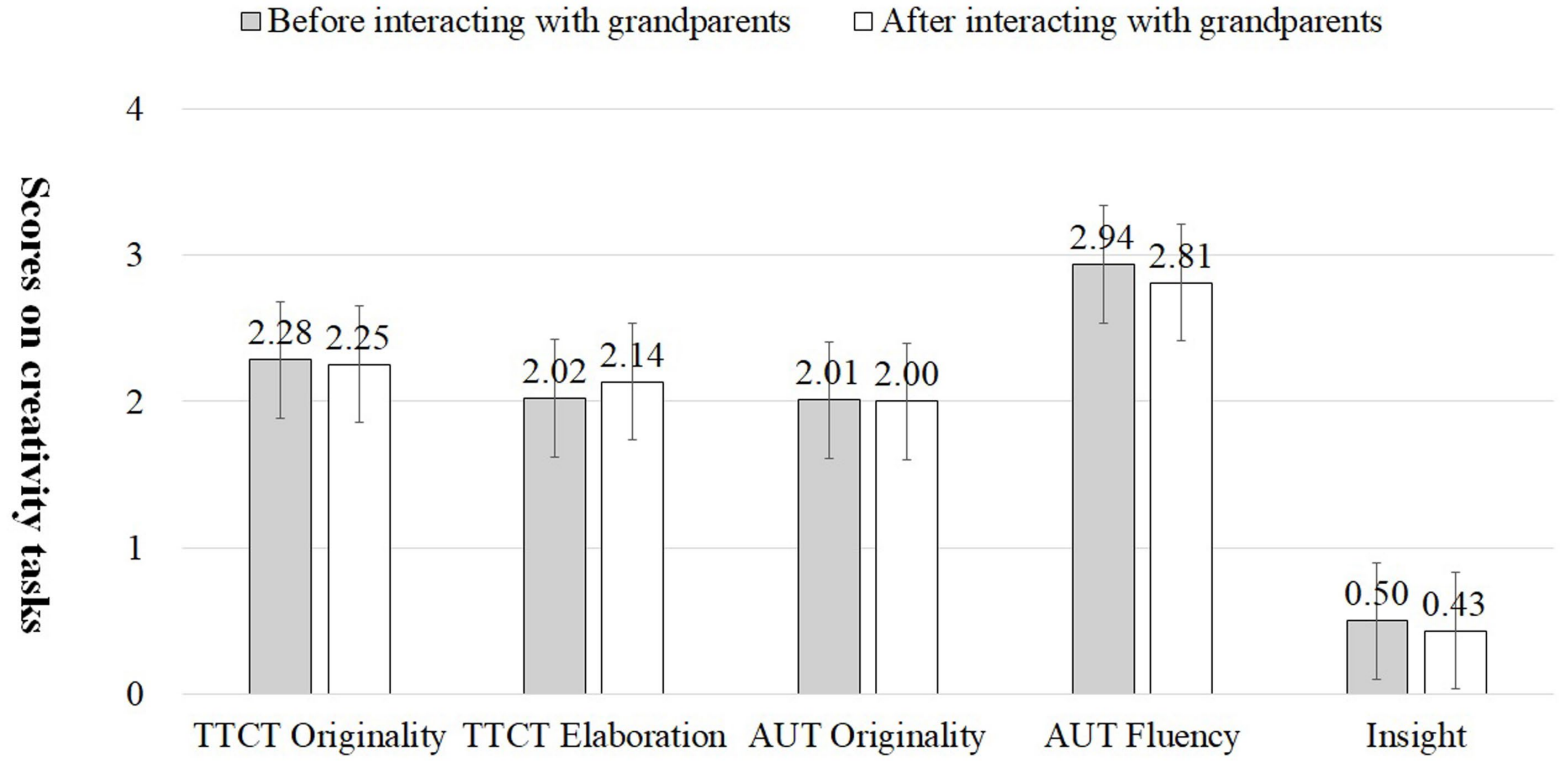
Differences in children’s scores on creativity tasks before and after interaction with grandparents.

Concerning whether adult-child interaction influenced children’s subsequent creative performance, repeated measures were conducted with “whether there was interaction” as the within-subjects variable and “which family member to interact with” as the between-subjects factor. The main effect of the two variables on the originality indicator of TTCT-Figural task is not significant, but the interaction effect is significant (*F* = 3.939*, *p* = 0.049). The interaction effect became nonsignificant when adults’ educational attainment was included in the analysis as a controlling variable. For the elaboration indicator of the TTCT-Figural task, the main effect of “whether there was interaction” was significant (*F* = 8.190**, *p* = 0.005), whereas neither the main effect nor the interaction effect of “interaction with which family member” was significant. Similarly, when we control for educational attainment in repeated measures, the main effect of “whether there was interaction” is no longer significant. Generally, the originality (*t* = 2.209*, *p* = 0.030) and elaboration (*t* = 2.984**, *p* = 0.004) of the TTCT-Figural task before and after parent–child interaction were significantly higher than those before parent–child interaction, but scores on the AUT and insight problem-solving tasks did not show significant differences before and after the parent–child interaction (see Figure 3; Table 5). However, only the elaboration of TTCT-Figural showed a significant difference before and after the grandparent–grandchild interaction (see Figure 4; Table 5). Hypothesis 2 is basically verified.

**Table 5:** Descriptive analysis of children’s creativity performance before and after interaction with adults.

	Creativity indicators	*N*	Interacting with parents		Interacting with grandparents	
			*M*	*SD*	*M*	*SD*
DT tasks	Children’s TTCT-Figural Originality (before interaction)	74	2.10	0.72	2.28	0.81
	Children’s TTCT-Figural Elaboration (before interaction)	74	2.31	0.81	2.25	0.84
	Children’s AUT Originality (before interaction)	74	1.95	0.74	2.02	0.75
	Children’s AUT Fluency (before interaction)	74	2.19	0.67	2.14	0.83
	Children’s TTCT-Figural Originality (after interaction)	74	2.03	0.64	2.01	0.77
	Children’s TTCT-Figural Elaboration (after interaction)	74	2.07	0.69	2.00	0.70
	Children’s AUT Originality (after interaction)	74	2.90	1.67	2.94	1.92
	Children’s AUT Fluency (after interaction)	74	2.93	1.70	2.81	1.60
CT tasks	Children’s insight problem solving (before interaction)	74	0.39	0.76	0.50	0.80
	Children’s insight problem solving (after interaction)	74	0.42	0.66	0.43	0.78

Furthermore, a correlation analysis was conducted between resources provided by adults in DT and CT tasks (i.e., cognitive resources and interpersonal resources), and children’s creativity performance, including TTCT-Figural originality, TTCT-Figural elaboration, AUT originality, AUT fluency, and insight scores, while controlling for the adults’ educational level (see Table 6). The results showed that the interpersonal resources given to children by adults were significantly related to children’s DT task performance. Whether pre- or post-interaction, children’s performance on TTCT and AUT was significantly correlated with adult-provided interpersonal resources, whereas the correlation between children’s creativity performance and adult-provided cognitive resources was not significant. There was also no significant correlation between children’s performance on the CT task and adult-provided resources. This implies that the more interpersonal resources provided to children by their parents or grandparents, the higher their performance in the divergent thinking tasks.

**Table 6:** Correlation analysis between resources provided by adults and children’s creativity performance (controlling for adults’ education levels).

	Resources provided by adult				DT task performance					CT task performance				
	1	2	3	4	5	6	7	8	9	10	11	12	13	14
1. Cognitive resources provided during children’s DT task	1													
2. Interpersonal resources provided during children’s DT task	0.153	1												
3. Cognitive resources provided during children’s CT task	0.689^**^	0.017	1											
4. Interpersonal resources provided during children’s CT task	0.141	0.676^**^	0.117	1										
5. Children’s TTCT-Figural Originality (before interaction)	−0.079	0.186^*^	−0.103	0.211^*^	1									
6. Children’s TTCT-Figural Elaboration (before interaction)	−0.078	0.117	−0.129	0.256^**^	0.617	1								
7. Children’s AUT Originality (before interaction)	−0.029	0.256^**^	−0.078	0.317^**^	0.317^**^	0.352^**^	1							
8. Children’s AUT Fluency (before interaction)	−0.064	0.168^*^	−0.146	0.198^*^	0.271^**^	0.336^**^	0.627^**^	1						
9. Children’s TTCT-Figural Originality (after interaction)	0.045	0.194^*^	−0.058	0.241^**^	0.568^**^	0.477^**^	0.246^**^	0.317^**^	1					
10. Children’s TTCT-Figural Elaboration (after interaction)	0.005	0.233^**^	−0.026	0.291^**^	0.467^**^	0.507^**^	0.301^**^	0.325^**^	0.564^**^	1				
11. Children’s AUT Originality (after interaction)	0.002	0.066	−0.042	0.143	0.260^**^	0.260^**^	0.638^**^	0.453^**^	0.232^**^	0.224^**^	1			
12. Children’s AUT Fluency (after interaction)	−0.005	0.084	−0.086	0.041	0.156	0.204^*^	0.448	0.636	0.237^**^	0.214^**^	0.666^**^	1		
13. Children’s insight problem solving (before interaction)	0.017	0.117	−0.067	0.107	−0.019	0.076	0.203^*^	0.131	0.046	0.118	0.105	0.028	1	
14. Children’s insight problem solving (after interaction)	0.126	0.153	0.035	0.160	−0.023	0.009	0.247	0.211	0.098	0.071	0.188	0.166	0.570	1
*M*	3.53	9.56	3.98	10.13	2.19	1.99	2.02	2.92	2.28	2.16	2.04	2.87	0.45	0.43
*SD*	3.45	1.95	4.12	2.51	0.77	0.74	0.71	1.79	0.82	0.75	0.70	1.65	0.78	0.72

** Indicates that the correlation is significant at the 0.01 level.

* Indicates that the correlation is significant at the 0.05 level.

## Discussion

Torrance and Hansen (1965) support that the family environment or interaction with parents may help to foster creativity in some way. Fearon et al. (2013) confirmed that the level of parental creativity is a fundamental predictor of children’s creativity levels and further emphasized the importance of children’s interaction with their parents. *The present study found that parents were able to provide more cognitive and interpersonal resources to their children compared to grandparents, thus influencing their children’s creative performance.*

Children’s creativity performance (mainly reflected in TTCT task scores) could be improved after interaction with their parents, while did not change significantly after interaction with grandparents, suggesting that it is not the “interaction with adults,” but “interaction with whom” that really matters children’s performance on subsequent creativity tasks. Interactions between children and adults may not contribute to creativity development in and of themselves; what really matters is the quality of interactions between children and adults. Why, then, do interactions with parents promote children’s creativity, but not interactions with grandparents?

Previous research has shown that both cognitive and emotional pathways may have an impact on children’s creative performance (Feldhusen, 2002; Runco, 2004). In the case of the cognitive pathway, the cognitive resources possessed by parents or grandparents may influence children through knowledge sharing (Lin et al., 2022). Simply put, knowledge sharing generated in interactions between adults and children may enhance children’s knowledge experiences in solving creative problems. Amabile (1988) demonstrated the potential influence of one’s task-related knowledge and expertise on one’s creativity. Knowledge can be viewed as a valuable resource that is distributed by individuals and becomes the property of the team as a result of sharing. Many scholars argue that knowledge sharing within teams provides opportunities for mutual learning, facilitates the creation of new knowledge, and enhances the ability of individuals to generate new ideas (Kogut and Zander, 1992; Tsai and Ghoshal, 1998). Successful knowledge sharing or knowledge exchange will allow teams to expand their knowledge base and combine existing knowledge to develop new solutions (Cohen and Levinthal, 1990).

We noted that the interaction effects of “whether there is interaction” and “interaction with whom” disappear when the level of education is included as a control variable. In fact, the increase in performance after the interaction may be attributed to the higher level of education of the parents. In general, there is a significant difference between parents and grandparents in terms of education level, which is also evident in the sample. Most parents had higher education (college or university degree, etc.), while grandparents’ education level was mainly elementary and middle school, with fewer grandparents with high school or higher education. Compared to grandparents, parents spend more time focusing on tasks in their interactions with their children. Their problem-solving skills and ability to discuss problem-solving solutions with their children are better than grandparents, which may serve as a better role model for their children. This also means that parents may have more valuable knowledge to share in their interactions with their children, thus increasing their children’s creativity. Existing research suggests that knowledge can increase the creativity of both sharers and receivers through three mechanisms. First, in the process of exploring the task, children gain a clearer understanding of the task they are doing. An individual may not be able to explain something clearly to his colleague unless he fully understands it, so knowledge sharing can develop into a good opportunity for individuals to expand their understanding of knowledge (Wang and Noe, 2010). Second, during the interaction, children can give feedback on the tasks they have completed in order to express them to adults, which will help them better understand the problems encountered, generate new ways to explain previous ideas, and integrate previous understandings into a new framework (Du Plessis, 2007). Third, in fact, when children interact with adults and complete knowledge sharing, there may be inconsistencies in perspectives and knowledge that may facilitate new ideas (Wang and Noe, 2010). Thus, interactions between children and adults are likely to lead to an exchange of perspectives and information, and this process of knowledge sharing will allow children to update their knowledge and skills (Perry-Smith and Shalley, 2003), and knowledge and experience are important prerequisites for generating creative ideas (Amabile, 1988). Although research confirms that parents provide more cognitive resources than grandparents during interactions with their children, the results of the correlation analysis showed that cognitive resources were not significantly associated with children’s creative performance. Therefore, it may be a factor that does not immediately affect creativity performance.

As for the emotional pathway, the results showed that the factor significantly associated with children’s creativity was the interpersonal resources provided by adults. According to the results of the study, parents have better emotional states during interactions with their children compared to grandparents, and children’s emotional states are positively correlated with adults’ emotional states. Previous research has generally concluded that positive emotions stimulate creativity more than neutral or negative emotions (Hirt et al., 2008). For example, inducing positive emotions has been shown to improve performance on the Remote Association Test (RAT), facilitate correct resolution of insight questions, and promote completion of divergent thinking tasks (Isen et al., 1987; Hirt et al., 1997, 2008). Several possible mechanisms have been proposed to explain this phenomenon (Hirt et al., 2008).

On the one hand, the positive emotions that adults and children bring to children may facilitate creativity by enhancing cognitive flexibility (De Dreu et al., 2008; Hirt et al., 2008). Cognitive flexibility refers to an individual’s ability to break old cognitive patterns, overcome functional fixations, and therefore make new (creative) associations between concepts (Guilford, 1967). For Guilford, flexibility (as opposed to rigidity) refers to the ability to consider new channels of thought. Guilford (1987) identified divergent production and transformation as the main categories in the Structure-of-Intellect Model for creative thinking. For open-ended problems (i.e., problems with multiple possible solutions), divergent production is often required (Kim and Runco, 2022). Previous research has shown that positive emotions help individuals to adopt flexible strategies when forming and testing hypotheses (De Dreu et al., 2008; Hirt et al., 2008), switch between information (Estrada et al., 1997), reduce anchoring effects during analysis and decision making, and improve executive functioning (Yang et al., 2013), among others, which contributes to changing the perspective of thinking, generating creative ideas, and the ability to find answers different from the conventional ones, which is especially important for creativity.

On the other hand, interactions between adults and children may have an emotionally motivating effect, thereby increasing children’s intrinsic motivation in subsequent tasks, which, in turn, influences children’s creative performance. Creativity research from the field of organizational behavior suggests that employees are more psychologically secure and more likely to speak up for new ideas when leaders show affirmation of the task (Tu and Lu, 2013). When leaders treat their employees with respect, it gives employees a higher sense of self-efficacy and strengthens their intrinsic motivation, thus promoting creative performance (Amabile et al., 1996; Tu and Lu, 2013). Therefore, the positive influence of adults on children’s creativity in the process of interaction may also be achieved through the good emotional state of parents, which promotes children’s intrinsic motivation. Furthermore, during interactions with children, although the verbal emotional support provided by parents (e.g., positive feedback, encouragement, etc.) did not differ significantly from that provided by grandparents, their physical proximity and closeness to children was significantly higher than that of grandparents. This unconscious physical or behavioral intimacy also reflected the closeness between parents and children, and nonverbal emotional support may be an important reason for enhancing children’s subsequent creative performance.

*The second finding of the study was that the positive effects of the interactions between children and adults occurred only in divergent thinking tasks, not convergent thinking tasks.* In general, researchers typically use either divergent thinking tasks (such as AUT) or convergent thinking tasks (such as insight problem solving) to measure creativity, but not both. In AUT tasks (e.g., “what are bricks for?”), participants need answer as multiple different ideas as possible (so it is divergent) to enhance the probability to make more novel output (Guilford, 1956). In contrast, insight problem-solving tasks point to a specific solution goal. Participants must get rid of the obstacles of familiar framework or existing rules under task constraints (Weisberg, 1995) in order to identify the correct answer. In this case, a successful solution requires not only divergent thinking, but also convergent thinking in the two-way process to build a novel and appropriate final solution.

In the last few years, researchers have explained the process of creative thinking from the perspective of dual-process theory (Stanovich and West, 2000; Ball et al., 2015; Althuizen and Reichel, 2016). The theory states that there are two types of processing in creative processes: the first type is default and unconscious processing, which is fast and automatic, while the second type is slow, controlled by consciousness, and requires more cognitive resources to engage in analytical processing (Evans and Stanovich, 2013; Lin and Lien, 2013).

DT and CT tasks can be distinguished by the “dual process” of creativity (Lin et al., 2012; Lin and Lien, 2013). Researchers have argued that divergent thinking relies primarily on Type I processing, which is responsible for intuitive associations, to obtain a large number of unique answers, whereas convergent thinking is an interactive process between Type I processing and Type II processing, which is responsible for analysis and evaluation, to determine the correct creative answer. Previous research supports this distinction, finding no correlation between individuals’ performance on divergent and convergent thinking tasks (Lin et al., 2005, 2012; Lin and Lien, 2013). Different types of emotions have different effects on the two measures of creativity: performance on divergent thinking can be improved by either positive or negative emotions, whereas performance on insightful problem solving can only be improved by positive emotions (Tsai et al., 2013).

The AUT and TTCT are commonly used in the measurement of divergent thinking (Runco and Acar, 2012), while insight problem solving is a typical for the measurement of convergent thinking. During children’s interactions with adults, the interpersonal resources provided by adults only had an effect on the divergent thinking task, but not on the convergent thinking task. It is possible that the interaction had an effect only on children’s intuitive processing and association (i.e., Type I processing), but not on children’s analytical thinking and evaluation (i.e., Type II processing). This study suggests that children’s emotional states were better when interacting with their parents than when interacting with their grandchildren, and it may be that it is this positive emotional state that enhances their subsequent divergent thinking performance.

Another possibility is that insight tasks are generally more difficult for children (especially those under third grade). In this work, children’s mean scores on the insight task were 0.45 (pre-interaction) and 0.43 (post-interaction), indicating that this type of creative task was too difficult for elementary school children, leading to poor results for the dependent variable across level conditions, suggesting a “floor effect” in the experiment. Comparatively, TTCT-Figural and AUT were more appropriate for the cognitive level of elementary school students in terms of difficulty, and children’s performance on these two divergent thinking tasks was more variable, with the dependent variable being more sensitive to the manipulation of the independent variable.

## Conclusion, implications, and limitations

First, children’s performance on divergent thinking tasks improved after interacting with parents, but not after interacting with grandparents. The interaction or main effect of the variables disappeared after controlling for adults’ education level. Comparatively, parents had much higher levels of education than grandparents, and the more educated the adults were, the more cognitive and interpersonal resources they could provide to children during the interaction. This suggests that adults’ educational attainment is an important factor influencing the quality of interactions with children, and that the quality of interactions has a positive effect on children’s subsequent creative performance.

Second, the adults-children interaction only had an effect on the subsequent divergent thinking task but not on the convergent thinking task, which may be that the interaction only facilitated children’s divergent thinking, or it may be that the convergent thinking measurement instrument chosen was too difficult. For younger age groups, creativity measurement instruments should be chosen more carefully.

Third, this study was unable to strip away the effects of SES on children’s creativity. Higher parental education was associated with higher quality parent–child interactions and corresponding increases in children’s subsequent creative performance. So, do children who raised by better-educated grandparents also enhance their creative performance after interacting with their grandparents? Or, is it possible to promote children’s creative performances by increasing the cognitive level of grandparents? Future research could be conducted further.

## Data availability statement

The raw data supporting the conclusions of this article will be made available by the authors, without undue reservation.

## Ethics statement

The studies involving human participants were reviewed and approved by the University Committee on Human Research Protection at East China Normal University (HR: 156-2018); furthermore, written informed consents were obtained from participants prior to study onset, and the methods were carried out following the approved guidelines. Written informed consent was obtained from the individual(s) and minor(s)’ legal guardian/next of kin, for the publication of any potentially identifiable images or data included in this article.

## Author contributions

YL conceived and designed the study. YL and YP performed the investigation. YL and WP conducted statistical analysis and the interpretation of article. All authors contributed to the article and approved the submitted version.

## Funding

Research supported by The Program for Professor of Special Appointment at Shanghai Institutions of Higher Learning (No.TP2020013).

## Conflict of interest

The authors declare that the research was conducted in the absence of any commercial or financial relationships that could be construed as a potential conflict of interest.

## Publisher’s note

All claims expressed in this article are solely those of the authors and do not necessarily represent those of their affiliated organizations, or those of the publisher, the editors and the reviewers. Any product that may be evaluated in this article, or claim that may be made by its manufacturer, is not guaranteed or endorsed by the publisher.
